# Drug-induced Leukocytoclastic Vasculitis Secondary to Trimethoprim/Sulfamethoxazole: A Case Report

**DOI:** 10.5811/cpcem.31056

**Published:** 2025-01-06

**Authors:** Ambika Shivarajpur, Simon Londono, Justin Shaw, Christopher Boccio, Leonid Melnitsky, Jheanelle McKay, Brian Kohen, Eric Boccio

**Affiliations:** * Memorial Healthcare System, Department of Emergency Medicine, Hollywood, Florida; † Florida International University, Herbert Wertheim College of Medicine, Miami, Florida; ‡ NYC Health + Hospitals/Bellevue New York, Department of Pharmacy, New York, New York; § Joe DiMaggio Children’s Hospital, Department of Pediatric Emergency Medicine, Hollywood, Florida

**Keywords:** leukocytoclastic vasculitis, Immunoglobulin A vasculitis, trimethoprim/sulfamethoxazole, drug reaction

## Abstract

**Introduction:**

Leukocytoclastic vasculitis (LCV) is a small vessel vasculitis typically affecting dermal capillaries and venules. The condition is often idiopathic but can be associated with infections, neoplasms, autoimmune disorders, and certain drugs.

**Case Report:**

A 91-year-old female with past medical history of Alzheimer dementia and hypertension, being treated for lower extremity cellulitis, presented to the emergency department for an allergic reaction. Trimethoprim/sulfamethoxazole (TMP/SMX) had been initiated six days earlier. The patient was noted to have normal vital signs. Palpable purpura was discovered on the lower back, buttocks, lower extremities, ankles, and feet. Laboratory studies were within normal limits. Given the clinical presentation, physical exam findings, and normal eosinophil count, the diagnosis of LCV secondary to TMP/SMX was made.

**Conclusion:**

Most cases of LCV are limited to cutaneous symptoms and self-resolve with supportive care.

## INTRODUCTION

Trimethoprim/sulfamethoxazole (TMP/SMX) is commonly prescribed for the treatment of various commonly encountered infections for numerous clinical indications. It is generally safe for immunocompetent patients; however, rare but potentially serious dose-unrelated adverse effects have been linked to both the TMP and SMX components. Gastrointestinal side effects including nausea, vomiting, and diarrhea are commonly reported in 3–8% of patients, with glossitis, stomatitis, and hepatotoxicity occurring more rarely. Cutaneous involvement ranging from maculopapular rashes to urticaria affect 3–4% of users; more severe reactions such as Stevens-Johnson syndrome are rare but may still occur. Trimethoprim/sulfamethoxazole has been associated with acute renal injury and hyperkalemia with higher rates of occurrence observed when administered at higher doses and in patients with renal dysfunction. Rare reports of hematologic conditions such as anemia and thrombocytopenia occur at rates similar to other sulfonamides. Psychiatric adverse effects including delirium and psychosis are observed at higher incidences in geriatric patients.^1^

Leukocytoclastic vasculitis (LCV) is a small-sized vasculitis characterized by inflammation and necrosis of arterioles, venules, and capillaries. The condition often presents as palpable purpura, which is typically confined to the lower extremities, although it may occasionally involve other areas. The etiology of LCV is diverse and has been associated with drugs, infections, autoimmune disorders, and malignancies. In a significant number of cases the cause of LCV remained unidentified. Leukocytoclastic vasculitis poses a diagnostic and therapeutic challenge, requiring careful consideration of potential triggers and management of possible complications. We present a case of drug-induced LCV secondary to TMP/SMX in an elderly female.

## CASE REPORT

A 91-year-old female with a past medical history of Alzheimer dementia and hypertension, undergoing treatment for bilateral lower extremity methicillin-resistant *Staphylococcus aureus* cellulitis, on day 6 of therapy with TMP 800 milligrams (mg)/SMX 160 mg orally twice a day, presented to the emergency department (ED) with a chief complaint of an allergic reaction. Per the skilled nursing facility, a rash was first noted on the bilateral lower extremities by the wound care nurse earlier that morning during routine dressing changes. The rash did not appear to be pruritic. Medication reconciliation revealed that the patient had also been taking carbidopa-levodopa 25–100 mg orally three times a day, digoxin 125 micrograms orally once daily, aspirin 81 mg orally once daily, and bisacodyl 5 mg orally once daily. The patient had no documented allergies, and no new known possible exposures to environmental allergens were reported. She was reported as being bedbound. The patient received 50 mg diphenhydramine intravenous (IV) from emergency medical services while en route to the ED.

Upon arrival, the patient was unable to provide further history due to advanced Alzheimer dementia. Initial vital signs were remarkable for mild hypertension (133/60 millimeters of mercury) but were otherwise normal (heart rate 67 beats per minute; respiratory rate 17 breaths per minute; peripheral capillary oxygen saturation 97%, room air), and the patient was afebrile (temperature: 36.5 °Celsius, oral). Physical examination revealed palpable purpura on dependent portions of the body, specifically involving the lower back, right upper extremity, bilateral buttocks, lower extremities, ankles, and feet (Image).

**CPC-EM Capsule**

What do we already know about this clinical entity?

*Leukocytoclastic vasculitis (LCV) is a small vessel vasculitis that typically affects dermal capillaries and venules*.

What makes this presentation of disease reportable?

*While most cases of LCV are idiopathic, it may present as an adverse drug reaction to trimethoprim/sulfamethoxazole*.

What is the major learning point?

*Failure to recognize the potential causes and manifestations of LCV often leads to missed or delayed diagnosis and unnecessary testing and treatment*.

How might this improve emergency medicine practice?

*Most cases of LCV are limited to cutaneous symptoms and self-resolve with avoidance of triggers and supportive care*.

Routine laboratory studies were performed. The complete blood count was remarkable for erythrocytosis (hemoglobin 15.8 grams per deciliter [g/dL] [reference range 12.1–15.1 g/dL]), leukocytosis (white blood cell count 13,200 cells per microliter (μL) [4,500–11,000/μL]), thrombocytosis (platelet count 530,000 platelets per μL, [150,000–450,000/μL]), and normal eosinophil count (absolute eosinophil count 31 cells per μL [30–350/μL]). The basic metabolic panel revealed an elevated serum creatinine compared to that of the last presentation two years prior (creatinine 1.10 mg/dL, increased from 0.51 mg/dL [0.6–1.1 mg/dL]). Given the clinical presentation, physical exam findings and normal eosinophil count, the diagnosis of drug-induced LCV secondary to TMP/SMX was made.

The patient was admitted to the hospitalist service for further monitoring, medical management, and non-emergent dermatology consultation. Trimethoprim/sulfamethoxazole was discontinued, and the patient was placed on minocycline 100 mg orally twice a day for seven days. Additional therapeutic treatment included methylprednisolone 40 mg IV twice daily, famotidine 20 mg orally once daily, and diphenhydramine 25 mg IV every eight hours. Dermatology was never consulted, and a skin biopsy was not performed. Per review of inpatient progress notes, the rash completely resolved on hospital day 6, and the patient was discharged on hospital day 7 on prednisone 10 mg orally once daily for five days. Outpatient follow-up at 96 hours revealed no sequelae, rash recurrence, or return visit to the ED.

## DISCUSSION

Vasculitis encompasses a spectrum of inflammatory conditions affecting blood vessels and adjacent tissues and is categorized by the types of vessels affected and the extent of localized or systemic involvement. With an incidence of 30 cases per million people per year, small vessel vasculitis is relatively rare and affects small blood vessels such as arterioles, venules, and capillaries.^2^ It can develop in association with factors such as infection, malignancy, autoimmune disorders, or drugs. Studies indicate that drugs account for approximately one-third of all cases of LCV, with non-steroidal anti-inflammatory drugs and certain antibiotics such as TMP/SMX frequently implicated. Antihypertensives, antiepileptics, immunosuppressive agents, hydralazine, and cocaine have also been reported to induce LCV, albeit less frequently.^3^

Leukocytoclastic vasculitis is characterized by inflammation of small blood vessels due to neutrophilic degranulation (leukocytoclasia) and subsequent infiltration, leading to hemorrhage, fibrinoid necrosis, and endothelial damage.^4^ The exact mechanism by which TMP/SMX induces LCV remains unclear, but current theories suggest a type 3 hypersensitivity reaction mediated by immune complexes. This process involves antibody-antigen complex deposition in small blood vessels, activating the complement cascade and recruitment of neutrophils, thereby inducing inflammation and vessel damage.^5^ Trimethoprim/sulfamethoxazole’s sulfur-containing nature has been theorized as the major contributor of its association with hypersensitivity reactions and has been linked to cases of Stevens-Johnson syndrome.^6^ Patient genetic factors, such as variations in the N-acetyltransferase 2 enzyme, may also influence susceptibility to TMP/SMX-induced LCV, necessitating further research.^7^

Most commonly, LCV presents clinically as cutaneous manifestations described characteristically as maculopapular and palpable purpura, often observed in gravity-dependent areas such as the distal extremities. Fever and generalized myalgias may also be present. More severe cases involve inflammation and restriction of blood flow to vital organs and tissues, resulting in systemic complications such as kidney failure and gastrointestinal and vascular hemorrhage. The diagnostic approach should involve a careful review of the medical history, a reconciliation of up-to-date medications, and a thorough physical examination. The differential diagnosis may include drug reaction with eosinophilia and systemic symptoms (DRESS syndrome), thrombocytopenic purpura, benign pigmented purpura, and Schamberg disease.^8^ Per the American College of Rheumatology, criteria for the diagnosis of LCV include age 16 years or older, recent initiation (1–3 weeks) of a new medication, palpable exanthem, and histopathology biopsy results demonstrating neutrophil infiltration of small vessel walls.^5^

The prognosis of LCV is favorable and related to disease severity as characterized by strict cutaneous involvement versus systemic involvement. The overall mortality rate of LCV is about 2%, and approximately 90% of patients experience resolution of skin lesions within weeks to months, while the remaining 10% average 2–4 years.^7^ The six-year survival rate is greater than 75% among all subsets of LCV. A large retrospective review of 112 patients revealed that 18% experienced relapse within 1–40 months following resolution of initial symptoms across all LCV subtypes.^9^

Several case reports have previously described TMP/SMX as a probable cause of drug-induced LCV. An 83-year-old female who developed purpura two days after starting TMP/SMX was found to have elevated cytoplasmic antineutrophil cytoplasmic antibodies titers, suggestive of small vessel vasculitis. A punch biopsy showed neutrophil and eosinophil infiltration of vessel walls as well as red cell extravasation consistent with LCV.^6^ Similarly, a 23-year-old male developed a rash on his foot three days after initiating TMP/SMX, which progressed to lower extremity purpura; histopathology was suggestive of small-vessel vasculitis.^5^ A 14-year-old female who was being treated for skin abscesses and oral ulcers developed purpura four days into her course of TMP/SMX with histopathology revealing perivascular inflammatory infiltrate with eosinophils and extravasation of red blood cells.^10^ Antibiotics other than TMP/SMX have been associated with drug-induced LCV. A 49-year-old female was administered ceftriaxone IV, which resulted in a violaceous rash on the lower extremities, and skin biopsy findings were consistent with LCV.^11^ All these cases are representative of uncomplicated LCV, involved only cutaneous manifestations, and were successfully managed with discontinuation of the antibiotic and initiation of steroid treatment.^6^,^7^,^10^,^11^ A single case report described probable TMP/SMX-induced LCV in a patient on chronic steroids, which was successfully treated with a burst of high-dose steroids.^5^ This case report suggests a possible dose-dependent relationship of steroids on LCV, which warrants further research.

## CONCLUSION

Clinical manifestations of LCV are typically cutaneous and are characteristically described as palpable purpura localized to dependent areas of the body. Most cases of LCV are idiopathic, present within 1–3 weeks of exposure, are limited to cutaneous symptoms, and self-resolve with supportive care. If an offending drug is suspected, discontinuation and avoidance are mainstays of treatment.

## Figures and Tables

**Image:**
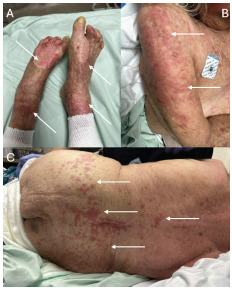
Typical cutaneous manifestations of leukocytoclastic vasculitis as evidenced by palpable purpura on dependent portions of the body including (A) bilateral ankles and feet, (B) right lateral upper extremity, and (C) sacrum, bilateral buttocks, and lower back (arrows).
